# Surgical Results in the Aisne Campaign

**Published:** 1918-09

**Authors:** 


**Surgical Results in the Aisne Campaign.** Tuffier, Acad. of Med. Feb. 5, Le Prog. Med., Feb. 23, states that the total mortality in the Zone of the Armies was 5.18%. The Ambulance troops had a mortality of 17.7%. (It is the duty of the medical profession to dissipate the popular notion that non-combatant troops of the Medical Dept. are seeking a safe service. Practically all statistics show that the actual risk is higher for the medical dept. for officers or enlisted men, than for the line). Mortality from shock has diminished. Gas gangrene (fatal and non-fatal) involved 3%. Tetanus 1: 2000. Abdominal wounds gave a mortality of 18% (formerly about 65%); thoracic 10.27%; cranio-cerebral 9.67%. Fractures have given remarkably favorable results, under the general technic of excision and immediate or delayed suture.

